# Supplementary material for: A Novel Multi-Target Small Molecule, LCC-09, Inhibits Stemness and Therapy-Resistant Phenotypes of Glioblastoma Cells by Increasing miR-34a and Deregulating the DRD4/Akt/mTOR Signaling Axis
Source: Cancers (Basel). 2019 Sep 26;11(10):1442. doi: 10.3390/cancers11101442 (PMC6826618; doi:10.3390/cancers11101442)
Supplement: Supplementary file 1 [file cancers-11-01442-s001.zip › cancers-578346-suppl 2.pptx]

## Slide 1
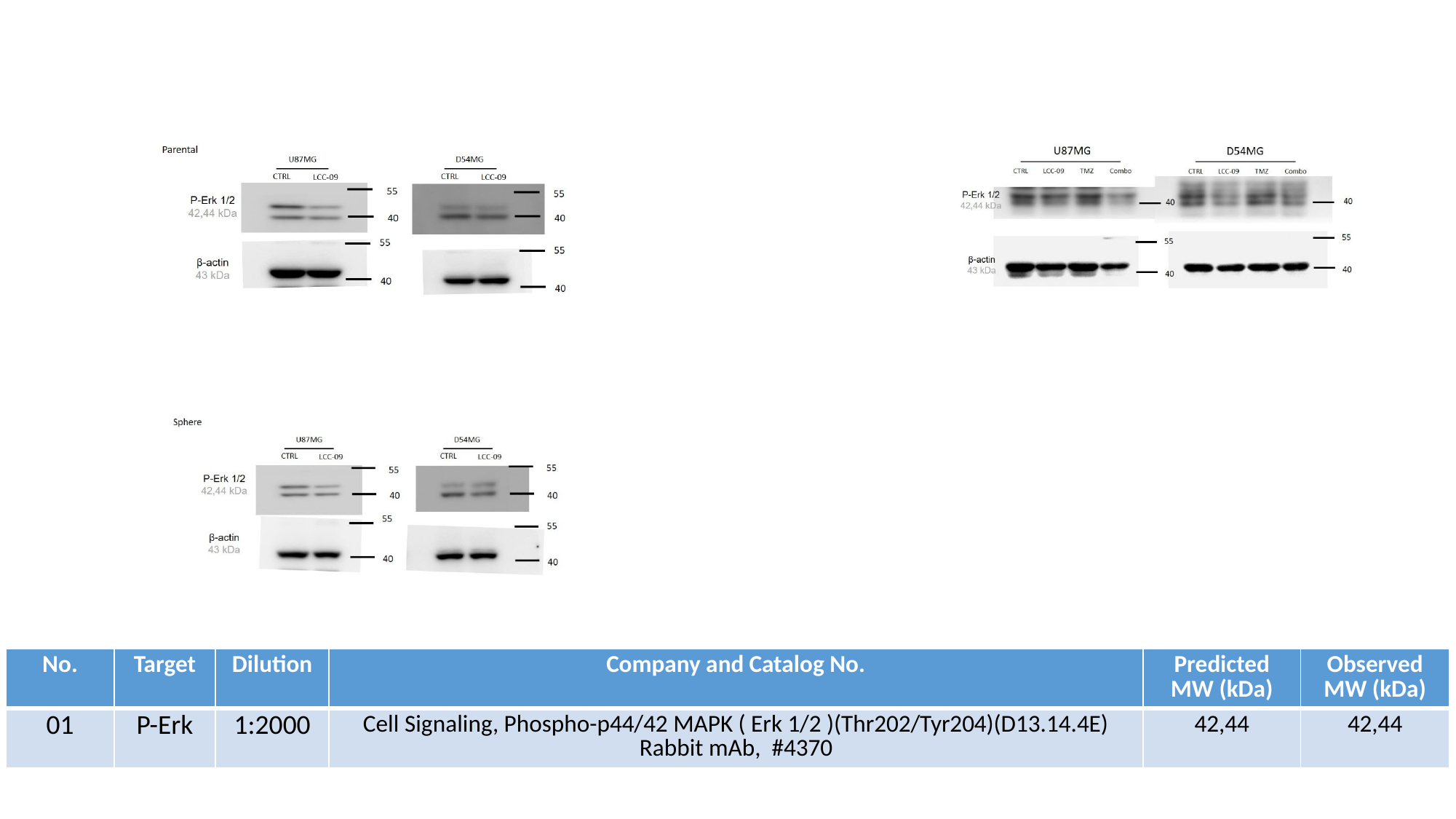

| No. | Target | Dilution | Company and Catalog No. | Predicted MW (kDa) | Observed MW (kDa) |
| --- | --- | --- | --- | --- | --- |
| 01 | P-Erk | 1:2000 | Cell Signaling, Phospho-p44/42 MAPK ( Erk 1/2 )(Thr202/Tyr204)(D13.14.4E) Rabbit mAb, #4370 | 42,44 | 42,44 |
