# Supplementary material for: A Novel Multi-Target Small Molecule, LCC-09, Inhibits Stemness and Therapy-Resistant Phenotypes of Glioblastoma Cells by Increasing miR-34a and Deregulating the DRD4/Akt/mTOR Signaling Axis
Source: Cancers (Basel). 2019 Sep 26;11(10):1442. doi: 10.3390/cancers11101442 (PMC6826618; doi:10.3390/cancers11101442)
Supplement: Supplementary file 1 [file cancers-11-01442-s001.zip › cancers-578346-suppl.pptx]

## Slide 1
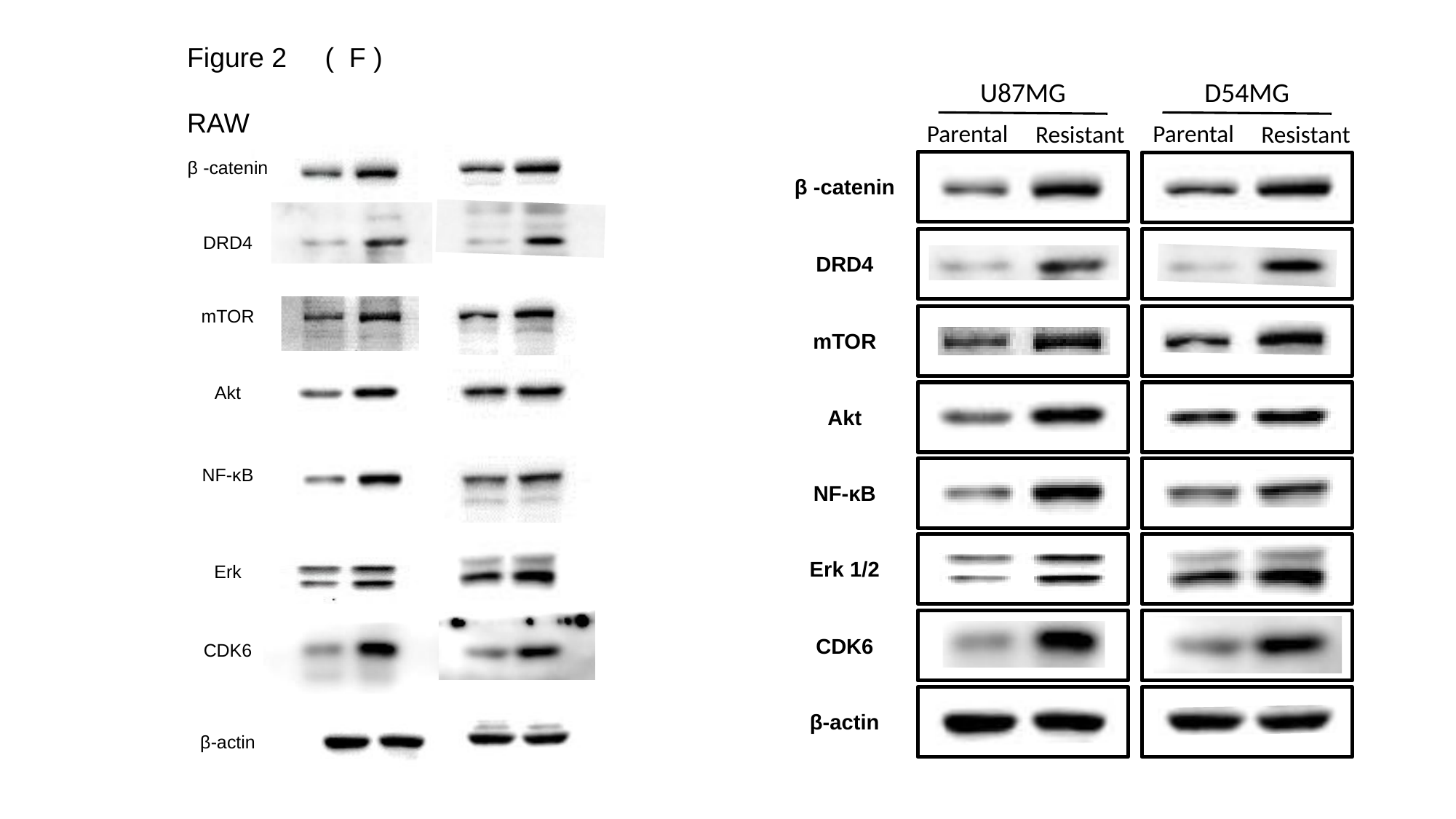

Figure 2 ( F )
RAW data
U87MG
D54MG
Parental
Parental
Resistant
Resistant
β -catenin
β -catenin
DRD4
DRD4
mTOR
mTOR
Akt
Akt
NF-κB
NF-κB
Erk 1/2
Erk
CDK6
CDK6
β-actin
β-actin

## Slide 2
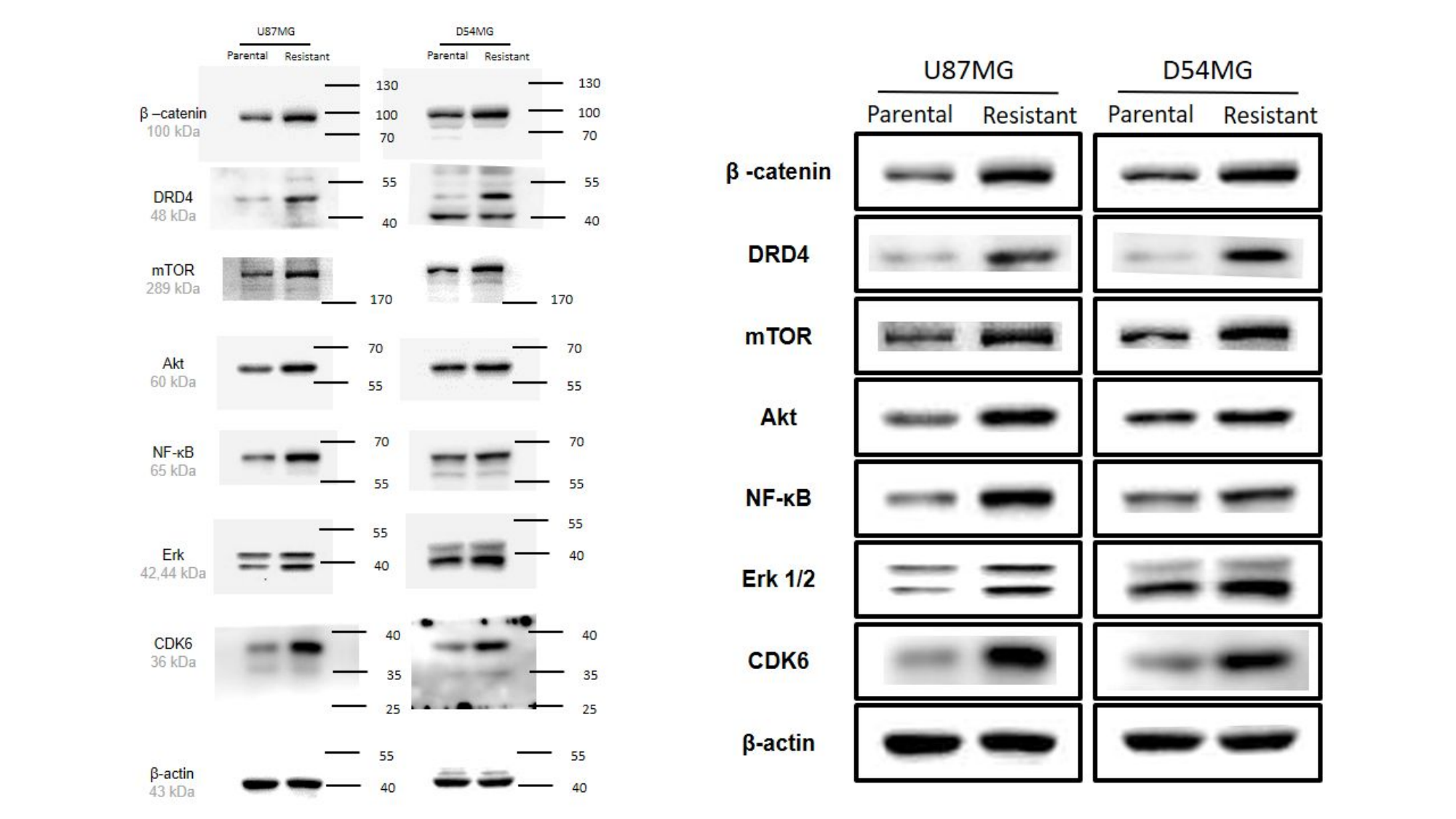

## Slide 3
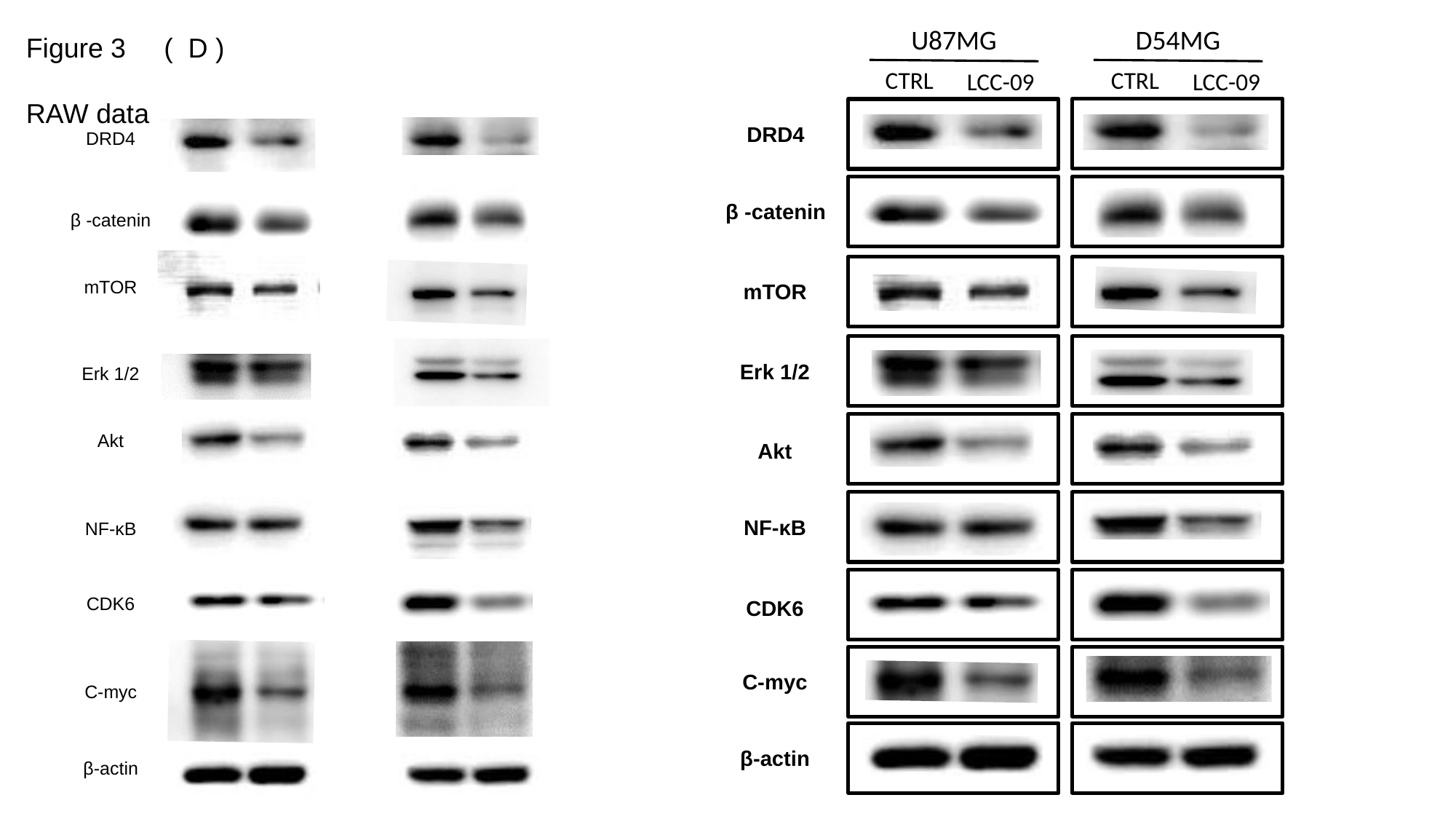

U87MG
D54MG
Figure 3 ( D )
RAW data
CTRL
CTRL
LCC-09
LCC-09
DRD4
DRD4
β -catenin
β -catenin
mTOR
mTOR
Erk 1/2
Erk 1/2
Akt
Akt
NF-κB
NF-κB
CDK6
CDK6
C-myc
C-myc
β-actin
β-actin

## Slide 4
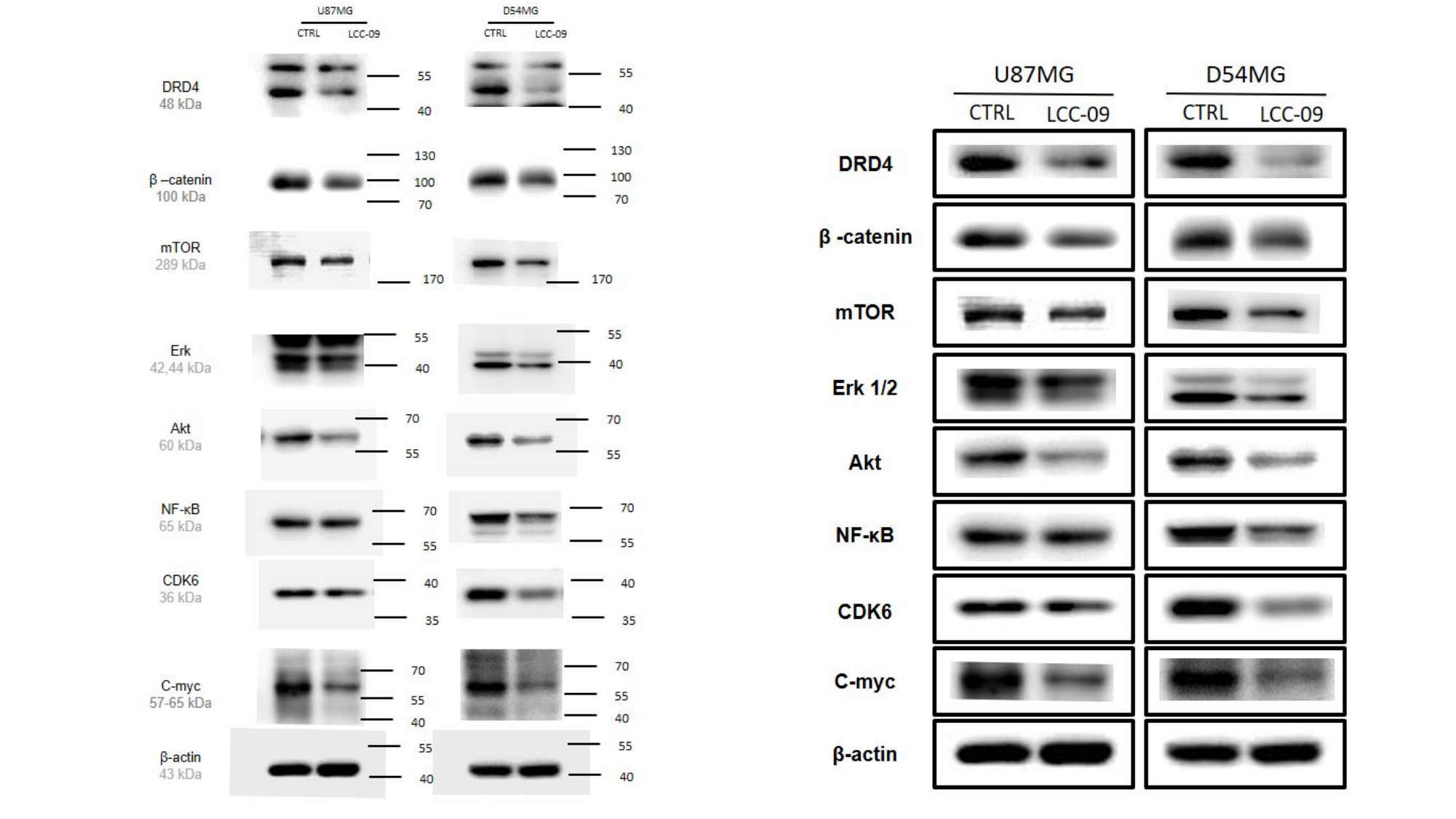

## Slide 5
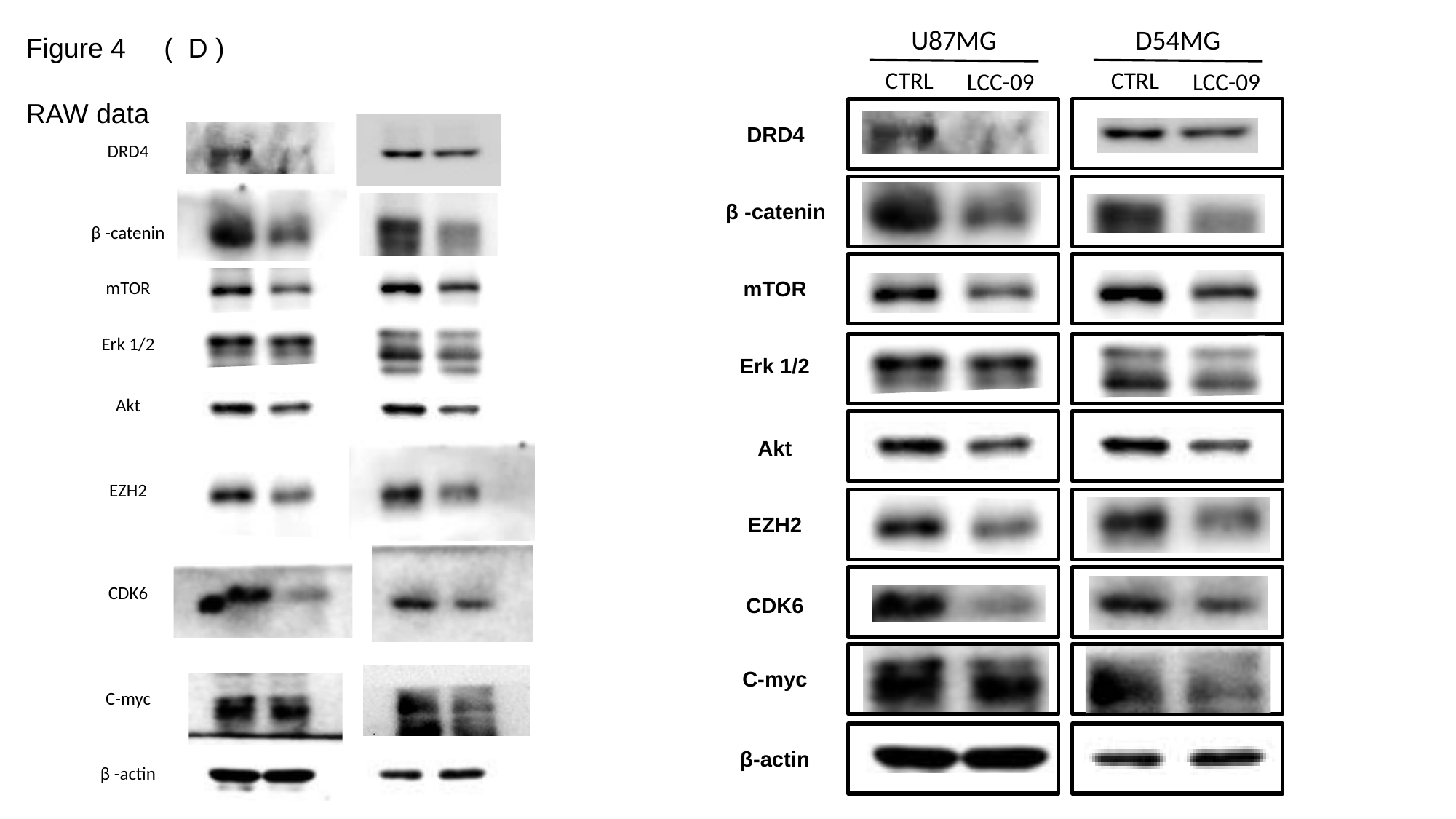

U87MG
D54MG
Figure 4 ( D )
RAW data
CTRL
CTRL
LCC-09
LCC-09
DRD4
DRD4
β -catenin
β -catenin
mTOR
mTOR
Erk 1/2
Erk 1/2
Akt
Akt
EZH2
EZH2
CDK6
CDK6
C-myc
C-myc
β-actin
β -actin

## Slide 6
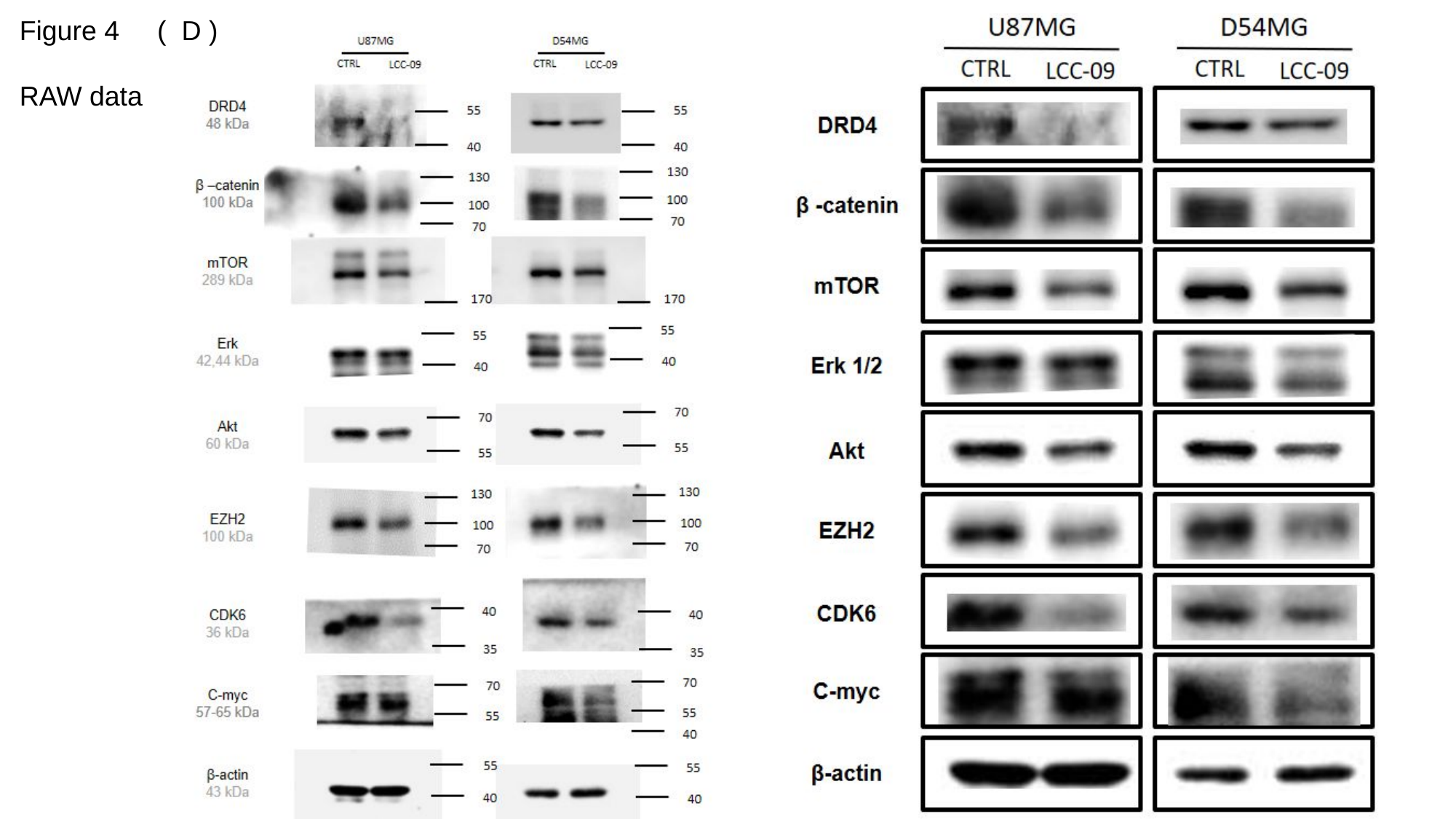

Figure 4 ( D )
RAW data

## Slide 7
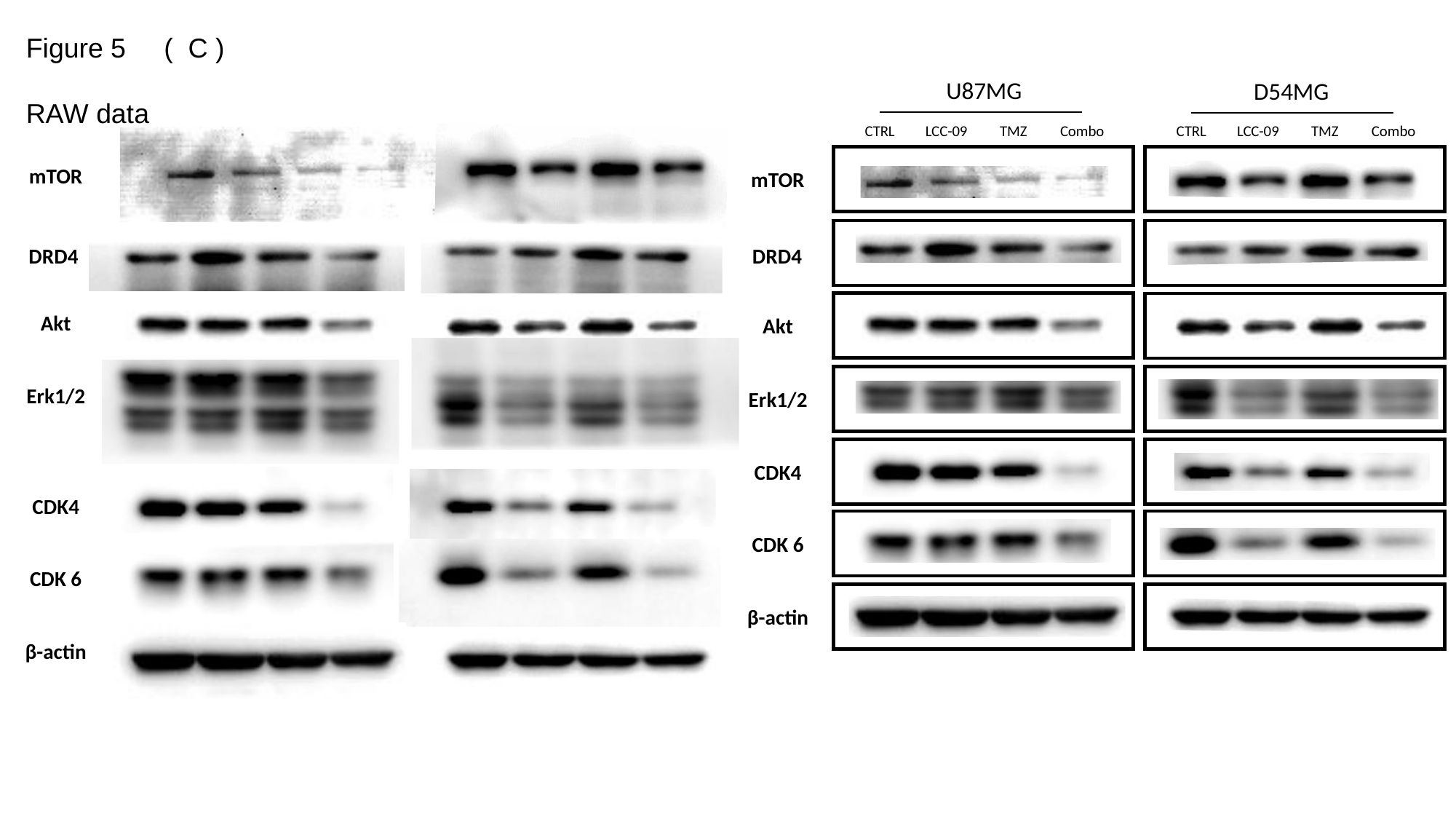

Figure 5 ( C )
RAW data
U87MG
D54MG
CTRL
LCC-09
TMZ
Combo
CTRL
LCC-09
TMZ
Combo
mTOR
mTOR
DRD4
DRD4
Akt
Akt
Erk1/2
Erk1/2
CDK4
CDK4
CDK 6
CDK 6
β-actin
β-actin

## Slide 8
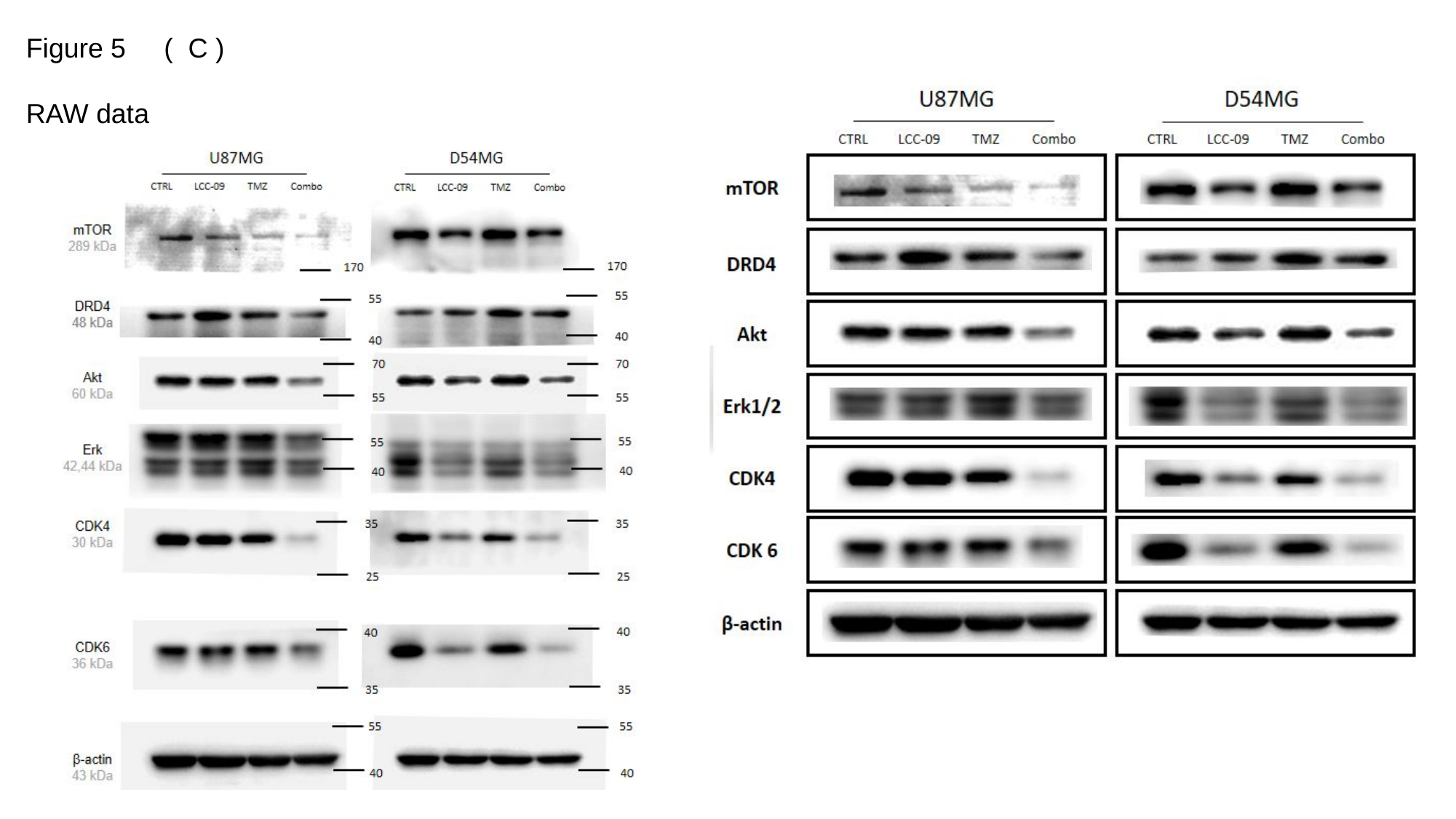

Figure 5 ( C )
RAW data

## Slide 9
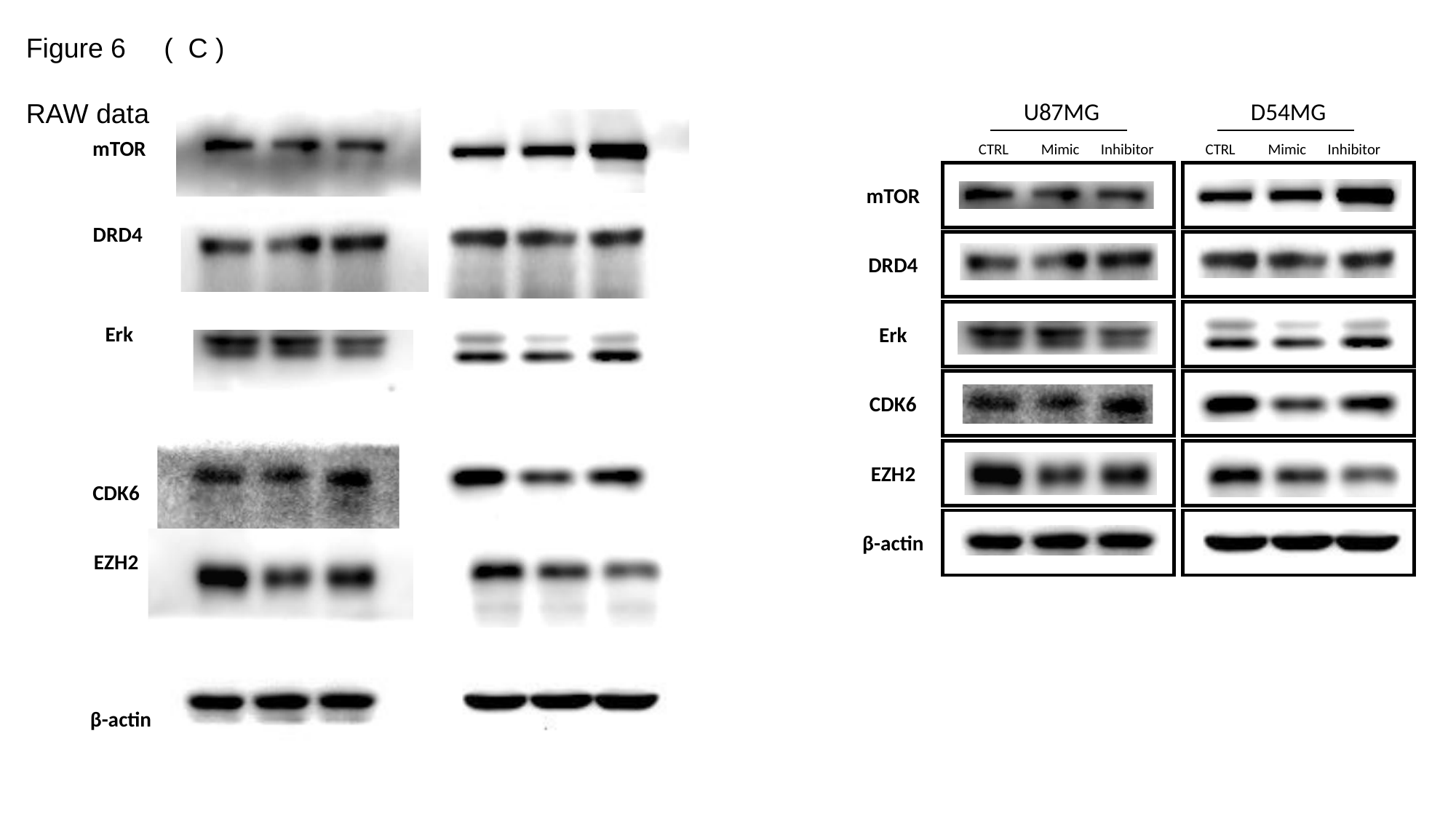

Figure 6 ( C )
RAW data
U87MG
D54MG
mTOR
CTRL
Mimic
Inhibitor
CTRL
Mimic
Inhibitor
mTOR
DRD4
DRD4
Erk
Erk
CDK6
EZH2
CDK6
β-actin
EZH2
β-actin

## Slide 10
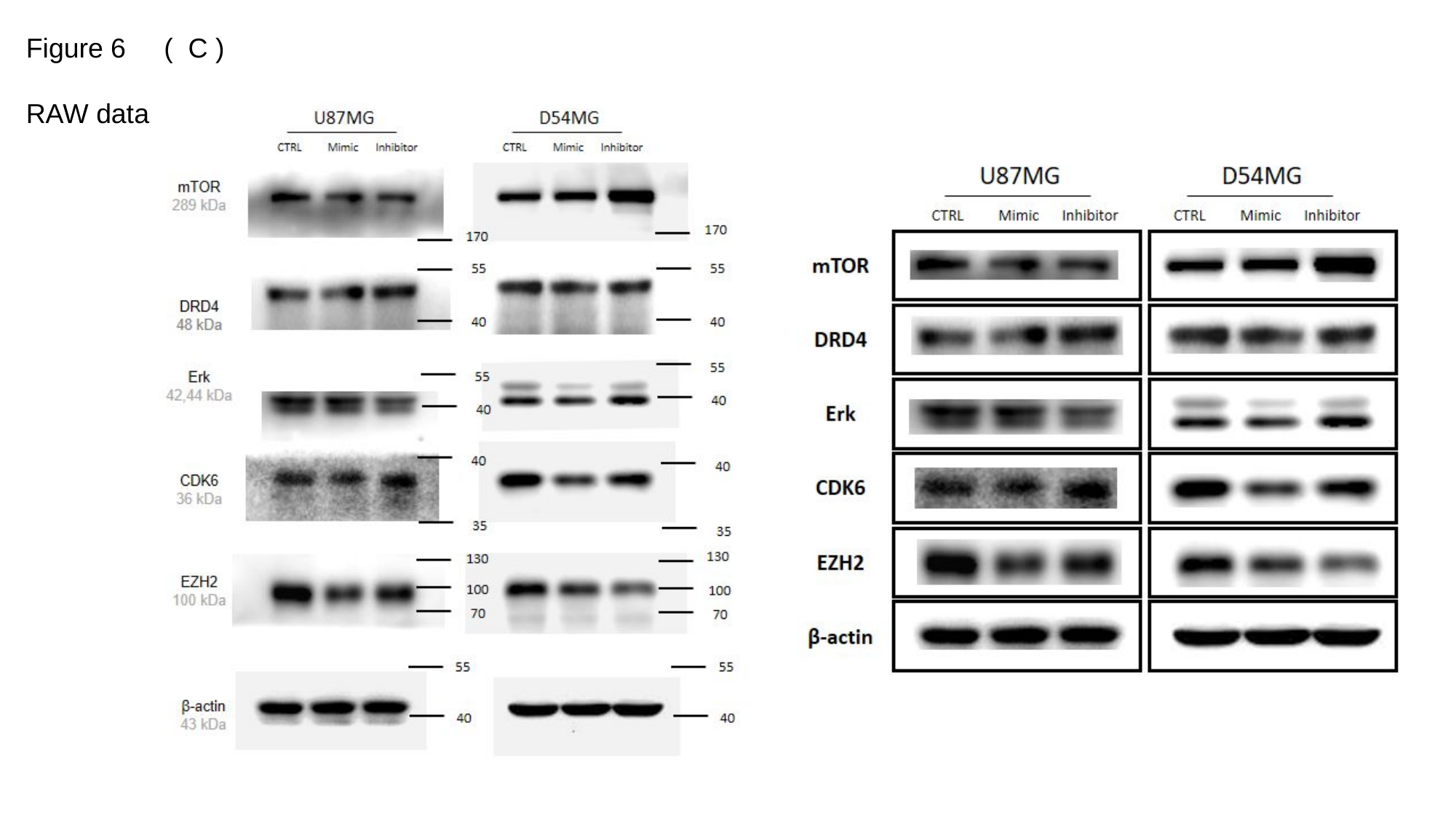

Figure 6 ( C )
RAW data

## Slide 11
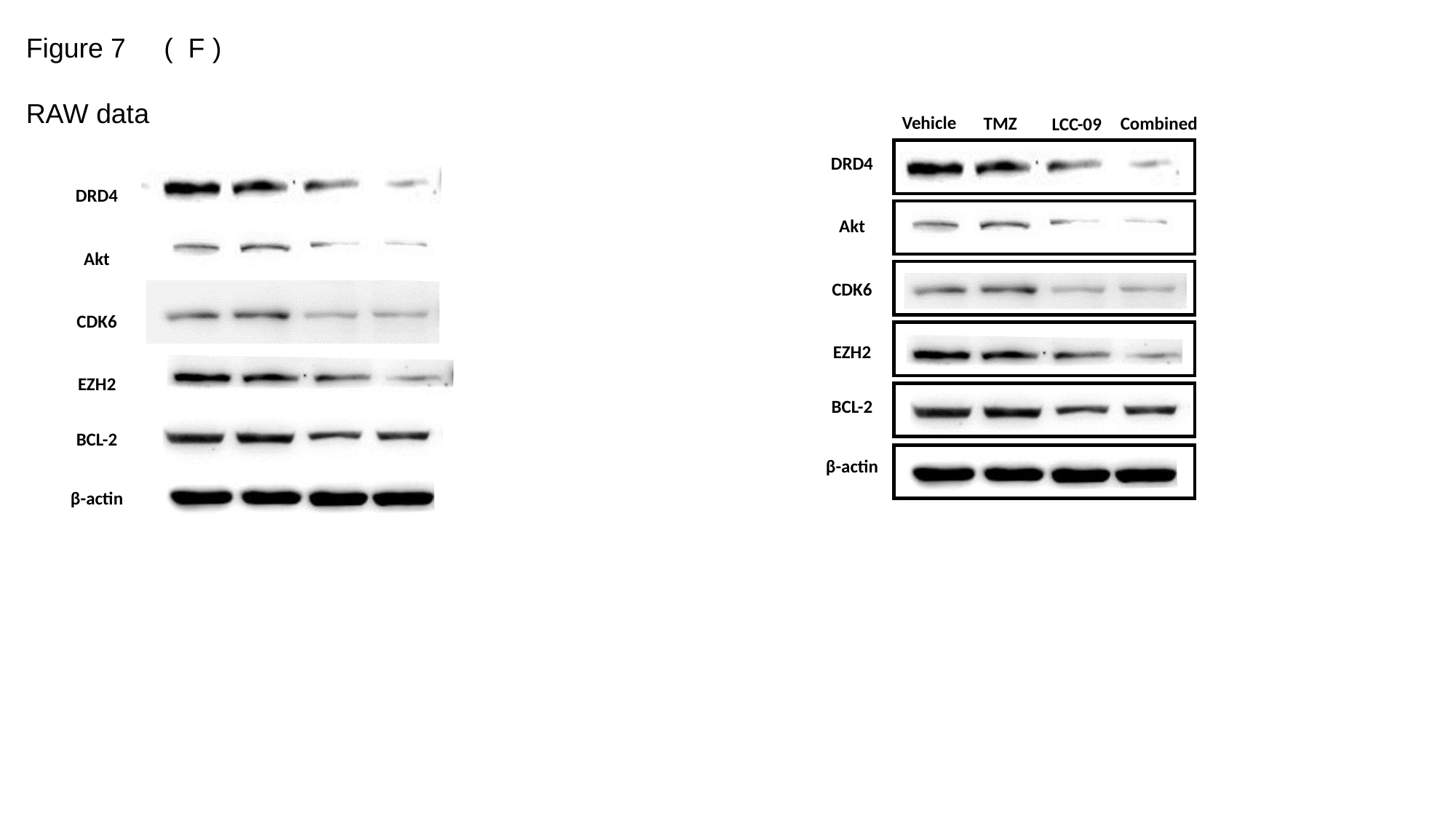

Figure 7 ( F )
RAW data
Vehicle
TMZ
Combined
LCC-09
DRD4
DRD4
Akt
Akt
CDK6
CDK6
EZH2
EZH2
BCL-2
BCL-2
β-actin
β-actin

## Slide 12
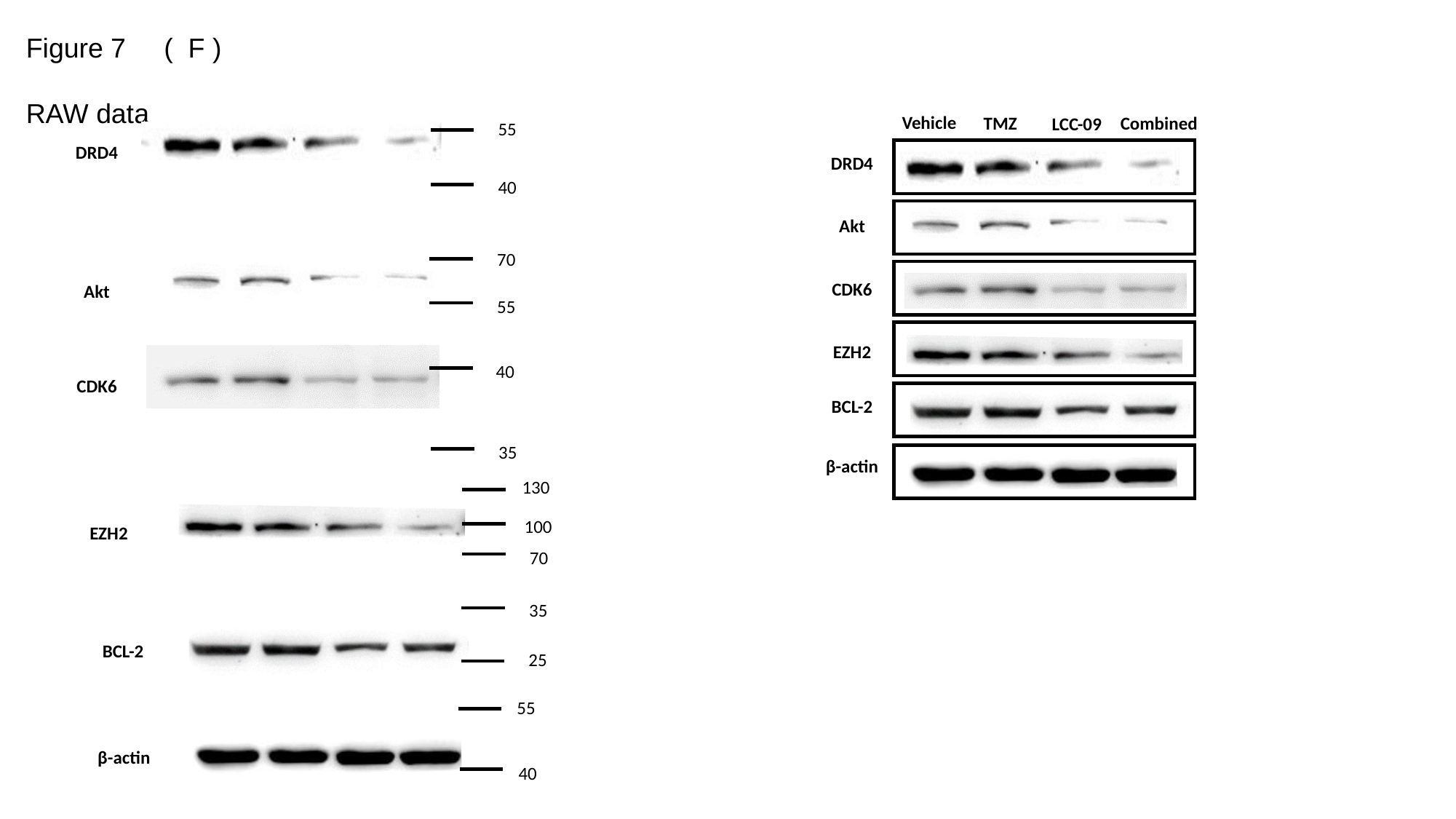

Figure 7 ( F )
RAW data
Vehicle
TMZ
Combined
LCC-09
55
DRD4
DRD4
40
Akt
70
CDK6
Akt
55
EZH2
40
CDK6
BCL-2
35
β-actin
130
100
EZH2
70
35
BCL-2
25
55
β-actin
40
